# Spatial spillover effects of skilled migration on innovation in China

**DOI:** 10.1016/j.heliyon.2024.e30849

**Published:** 2024-05-07

**Authors:** Xing Gao, Jin Zhu, He Zhu, Xingman Zhang

**Affiliations:** a School of Economics, Beijing Institute of Technology, Beijing, China; b Department of Real Estate and Construction & Department of Urban Planning and Design & Urban Systems Institute, Faculty of Architecture, The University of Hong Kong, Hong Kong SAR, China; c School of Architecture and Urban Planning, Beijing University of Civil Engineering and Architecture, Beijing, China

**Keywords:** Skilled migration, Innovation, Spatial spillover, Change, China

## Abstract

Despite the importance of interregional skilled migration to regional development, few studies have explored its spatial spillover effects and their changes over time. Thus, employing the Spatial Durbin Model, we investigate the presence of regional spillovers of skilled migration at both national and sub-national levels in China. Especially, we focus on the regional difference and change in the spatial spillover. Although our results confirm positive spillover effects at the national level due to the strong mobility characteristic of skilled migrants, developed regions benefit more from spillovers of skilled migration than developing regions, and such effects are divergent in different regions over time. Our findings also indicate that changes in spatial spillovers among regions are closely associated with the mobility of economic factors in geography. Theoretically, by considering the spatial effects of skilled migration on the innovation output of recipient regions, we extend the labour economics literature into geographical economic agglomeration, especially innovation economic geography. Methodologically, we examine the spatial effects at both national and sub-national levels, and capture the spatial externalities; we also apply Maximum Likelihood estimation to assess the endogeneity issues to understand the mechanisms of spillover change over time. The study can be of significance for municipalities in the policy-making of attracting talents and promoting regional innovation.

## 1 Introduction

Skilled migration is an important measurement of the diffusion of technology, knowledge or innovation in recipient areas. As sources of valuable tacit knowledge, skilled migration mediates local economic actors by creating a contextually enabling environment for innovation. The environment is promoted by the externalities linked to human resources capital accumulation [1]. Also, skilled individual mobility is usually intertwined with regional inequalities. Therefore, exploring the distribution of skilled individuals and spatial effects can evaluate the opportunity equalities in regional development, which is associated with long-term economic growth and societal stability. Within the same geographical context, the coexistence from different sources of individual embodied, tacit knowledge promotes the localised process of cumulative learning, which would improve innovation capabilities in host regions [2]. In this article, skilled migration focuses on the spatiality of regional skilled individuals’ mobility.

Since the reform and opening-up, China's remarkable growth is accompanied by the abundant domestic migration [3] but is also featured by widening gaps in regional economic development and innovation. In practice, China has implemented subsequent regional policies to support the development of Central and West China [4] and introduced some incentives to attract talents in these underdeveloped regions to counteract economic and innovation inequalities. Nevertheless, the change and the long-term effects of skilled migration on innovation capabilities are understudied. Simultaneously, existing studies have recognised that regional inequalities and the changes are sensitive to spatial scale [5,6]. After the tax-sharing reform in 1994 and the subsequent economic decentralisation [7], local states in China have held substantial power in making local policies to attract skilled migrants and facilitate innovation growth.

Previous studies have extensively examined the influence of skilled migration on regional growth and the spatial patterns and determinants of skilled migration [1,8,9]. While the positive associations between skilled migration and economic growth are commonly accepted, whether significant spillover effects exist across regions remains unanswered. Specifically, due to the difference in spatial scales, is the effect of skilled migration on innovation at the national level higher than that at the sub-national level? Also, have the influences caused by skilled migration extended beyond the limits of a region? To the best knowledge of the authors, scant research testified regional spatial spillovers from skilled migration. The reason may be that finding a case where its territory is disaggregated into some regions with substantial political powers is difficult [10,11]. Moreover, when the spatial patterns and the determinants of skilled migration are considered, the spatial analysis in the existing studies only focuses on the first-round neighbours whose purpose of a specification is linear in the parameters, and the spatial externalities and endogeneity issues are ignored. In addition, current studies on spatial effects of skilled migration did not capture multi-level spatial scales, which is important for region-specific planning and policy-making.

This study aims to investigate the presence of regional spillovers of skilled migration at both China's national and sub-national levels. Especially, the study underlines the mobility of skilled migration at different spatial scales and its regional difference in the spatial effects. By employing Spatial Durbin Model (SDM), we address the following two questions related to skilled migration geographically: (1) Does spatial spillover of skilled migration to regional growth exist across regions; and (2) if yes, how does the spillover effect change over time in different regions. Thus the study has the following significances. First, we map the spatiotemporal patterns to show the regional difference in skilled migration. Second, we examine the spatial effects and mechanisms of spillovers change of skilled migration to regional innovation output, rather than GDP. This helps us deepen the thinking on labour capital externality and agglomeration economy in the literature of innovation economic geography. Currently, the studies focus too much on the economic performance of skilled immigrants, from the view of labour economics, and the effects of employment and wages [12,13]. Few, if any, have explored the internal migration ﬂows and regional innovation performance within the field of economic geography. Third, the study not only considers the first round neighbours but also captures the spatial externalities by including spatial autoregressive and spatial error models at both national and sub-national levels, which will offer a more complete and accurate estimation on the spillover. In addition, endogeneity issues also will be solved by Maximum Likelihood estimation.

## 2 Literature review

### 2.1 Skilled migration in China: from urban-rural dual system to “knowledge turn”

The current studies relatively explore macro level urban-rural dual system and corresponding institutional and governance models at macro level [14]. Especially, in the process of China's transition from a planned economy to a market economy, these studies discuss the impact of national institutions and governance on domestic migration [15,16]. This system is a guarantee of urban citizenship and social rights. Migrants who are able to settle permanently are mostly influential social and economic elites, while temporary immigrants are mainly vulnerable groups [3]. Thus, a large number of cheap labour have emerged in developed regions. Although the regional economy has developed rapidly, it has also led to many urban problems, such as housing, social security, healthcare, and education [17]. These have lowered the cost of labour reproduction for immigrants and increased the cost of urban governance [16]. Furthermore, modern world is a society where space is not fixed, stagnant, and rapidly flowing. Especially, with the development of China's market economy, knowledge or innovation has become the dominant force for regional growth [18]. The impact of a large amount of cheap labor on regional economies is gradually decreasing. Therefore, although the urban-rural dual system and structure have important analytical power in understanding domestic migration, it has not yet fully paid attention to how immigrants can actively achieve growth in their immigration areas through knowledge practice and build new social innovation networks in the Chinese context. The “knowledge turn” has opened up a new perspective for understanding how geographic immigration policies and structures shape and transform into regional innovation and growth. In other words, knowledge can only be placed in specific immigration systems and rights protection processes to achieve sustainable economic growth. This also means that immigration geography is transitioning from a macro structural analysis perspective to a knowledge centered micro spatial perspective focusing on sustainable growth at the subnational level, and emphasizes the role of skilled migration in actual spatial growth.

Similarly, the knowledge-based transformation of China's economic development history, to some degree, a story of migration. In the past, China adopted an extensive economic growth model, and a large amount of cheap labour input was one of its main characteristics [19]. The economic growth achieved through this approach has the consequences of higher consumption, higher costs, lower product quality, and lower economic benefits [20]. However, in the age of knowledge-based economies, the agglomeration of highly specialized services and command functions in China's global cities demands a large number of highly skilled labour resources, which attracts a large pool of skilled workers from other places [21]. In particular, with China's market economy reform, innovation has gradually been developed into a national priority strategy by effectively absorbing and applying all kinds of knowledge and technologies. Thus, various preferential policies have emerged in China's regions and cities to attract talents, such as housing, living subsidies, education, medical care, etc. [22]. “Knowledge turn” has begun to disintegrate the traditional regional migration model. On the other hand, due to differences in geographical location and immigration policies, the divergence of regional economic performance is also driven [23]. Although the emergence and mobility of high-skilled labour was driven by political and administrative forces in the early stage of China's reform and opening up, the prosperity of the secondary industry, mainly manufacturing, has also increased the number of skilled workers from 1980 to 2000. Especially, technology and knowledge have propelled the management and historical evolution of manufacturing [24], indicating that the development of manufacturing has is closely related to skilled labour. Furthermore, current literature has indicated that the influx of low-skilled immigrants can lead to the decline of manufacturing [25,26].

From 1990 to 2004, China's higher education system provided skilled labour with the widest range of opportunities in the job market, which makes “knowledge turn” become the leading force in the growth of regional migration. Because the public education system alone cannot meet individuals' demand for high skills, private higher education is rising rapidly, especially the majors with high returns such as business and information and communication technology [27]. Higher education institutions usually influence regional growth by the quality increase of workforce visible in the labour productivity [28], and the increasing workforce quality facilitates the spatial diffusion of innovation and ideas, as well as the sustainable regional economic growth [29]. For example, the proliferation of white-collar jobs is accompanied by urban economic structural change which requires more skills and knowledge. Although the system is viewed inclusive and widespread, the location of higher education and graduate employment seems to result in regional inequalities [30]. The intensity of human capital in a region is determined by regional specialisation based on relative factors endowments [31]. Thus, the number and scale of skill-intensive commercial towns are increasing, such as Beijing, Shanghai, Shenzhen, etc. It means that the growth of cities and towns depends on technological innovation and knowledge agglomeration, and the thinking of entrepreneurship is integrated into urban growth [32,33]. In order to achieve this goal, a series of local policies to attract talents have emerged, including subsidies, rewards, housing, etc [34,35]. At the same time, the diversification of production bundling is realised by providing valuable knowledge-intensive services. This process prompted highly paid and highly skilled employees to move to the region, eventually improving their living standards.

After 2004, dramatic industrial upgrading and economic restructuring are rapidly unfolding in China [36], which requires extremely high demands for technological innovation. Thus, “knowledge turn” perspective shows the transformation of the employment structure shifting from low-skilled towards skilled labour has been gradually obvious and mature. However, the talent or skilled labour alone is not equal to the innovation or economy growth, and cannot polish up other economic shortcomings, such as bureaucracy, academic dishonesty etc [37]. Especially, regional differences in skilled migration have been further widened. With the rapid development of commerce and high-tech industries in eastern coastal areas, the demand for professionals is growing rapidly, whereas the central and western regions with weak economic status have failed to retain and attract such skilled labour [9]. In addition, the further expansion of higher education and the development of international education have also saved a lot of skilled labour for technology-intensive industries [38]. Until now, the analogy between the knowledge economy and China's growth is perceived not only in the rise of skilled labour, but also by the unprecedented evolution compared with any other region. Thus, China can be regarded as a skilled migration paradigm to explore the increasing inequalities and spillover within the country.

### 2.2 Theoretical discussion

Developing countries have paid growing attention to that the cluster of economic activities enables different types of skilled labour to participate in different forms of interaction, thus stimulating more activities [39]. Due to the externality of knowledge and information flow, regions can learn from each other through network and knowledge spillover effect, so as to stimulate innovation and economic growth [40]. Thus, the spillover is viewed as the transition by physical interactions between knowledge holders (skilled migration), which emphasizes the importance of geographical proximity. Therefore, localisation economies and urbanisation economies can help to understand the spillover of skilled migration. Localisation economies mean the benefits linked to the spatial concentration of economic activities within a given industry, and urbanisation economies refer to the scale economies caused by the concentration of economic activities, regardless of industrial composition [41]. The two kinds of economies seem to be being discussed in an urban or developed regional context because of the dependencies between different types of cities or regions [42]. Based on the interaction between intra-regions and inter-regions with different development levels, skilled labour would be more potential for sharing, matching and learning new knowledge. Especially, for less-developed regions, skilled labour may have more to gain from increasing agglomeration, because compared with developed regions, the marginal effect of increasing scale and diversity of skilled migration would rise [43]. Thus, although the spatial distribution of new science-intensive industries seems to be systematically associated with the geographic distribution of skilled human capital in developed regions [44], spatial interactions in skilled labour are still important. This is because the mobility of highly productive inventors is a key channel of positive knowledge externalities [45].

Increasing shares of knowledge-intensive labour result in higher need for knowledge-intensive and regular industries [46]. Meanwhile, evidences from agglomeration economies also indicate that skilled labour spillover in cross-industry is more significant in less-developed regions [47]. Thus, skilled labour can promote the production of new knowledge in different industries and regions. A region can begin the virtuous circle when it locates near highly skilled labour pool and benefits from knowledge spillovers. Further, the price of skilled employment rises and spatial agglomeration will be deepened [48]. Therefore, Jacobs [49] argues that a diverse skilled labour structure contributes to the interactions between regional regions and the knowledge combination in various industries, which improves the innovative and imitative potential. On the other hand, valuable skills tend to be geographically bound. Highly skilled individuals cannot move completely in space, and their interaction is still limited by the specific spatial environment [1]. The increase of existing knowledge stock promotes the motivation of collective learning, which is enhanced by the externalities related to the process of skilled human capital accumulation. In a given geographical space, the relational networks consisting of individuals, industries and governments with different knowledge bases can support innovation generation and transfer [50]. In addition, due to the existence of internal and external learning, local capabilities are related to speciﬁc spatial and industrial environment and their neighbours [51]. Thus, skilled migration can affect a region by increasing local human capital stock and the creating a ‘contextually enabling environment for innovativeness’ [52].

Meanwhile, international evidences have also indicated the spillover effect of skilled migration to knowledge accumulation because they directly participate in knowledge-generation activities [53,54]. Therefore, skilled migration is considered as a main motivation to drive the innovation and knowledge diffusion at different spatial scales [55,56]. In theory, Marshall [57], Lucas [58] and Acemoglu [59] argue the positive spillover of skilled migration in a certain space. They suggest that the positive spillover effect is achieved by knowledge diffusion and implementing new technologies with a limited space. In addition, the theory of diversity also illustrates the spillover contribution of skilled migration to knowledge diffusion and economic growth [60]. Furthermore, high mobility will be accompanied by large spillover effects in a region, and the positive effect of diversity by skilled migration would increase over time [61]. These theories are also supported by empirical literature. For example, the positive spillover effect of skilled migration will decrease with increasing distance [62]. The inflow of skilled migration will have a gradually increasing positive impact, but the corresponding outflow will have a more or less sudden negative impact [61].

However, this spillover effect is not always positive and there are geographical differences. For instance, according to the US experience, the combination of migration policy and self-selection on technical and engineering skills encourage skilled migration to produce improved knowledge and greater skills [63]. Moreover, the effect in the US is always positive because crowding-out effects on natives cannot work [64]. On the contrary, due to the different nature of skilled migration, the evidence from Europe shows mixed results. The uneven spatial distribution of skilled migration in OECD makes the positive spillover effects of skilled migration be challenged [65,66]. In addition, the negative spillover effect of skilled migration on knowledge or innovation may be from diversity, especially cultural and ethnic diversity, because diversity can create obstacles to interaction and communication and lead to conflicts and a lack of joint action [67]. If the abilities and experiences of immigrants are extremely disconnected, then innovative learning will also be more difficult. whether labor market conditions and demand in different regions can complement skilled migration also determines whether there is a positive spillover effect [65,68]. Overall, geographical proximity is a necessary condition to take advantage of the benefits brought by the increase of human capital stock and stimulate the emergence of valuable externalities. Moreover, regional heterogeneity remains a key perspective in understanding how the increased availability of new knowledge caused by skilled migration result in innovation output. Consequently, we explore the spatial spillover and its change mechanisms of skilled migration.

## 3 Methodology

### 3.1 Case study

**Fig. 1:**
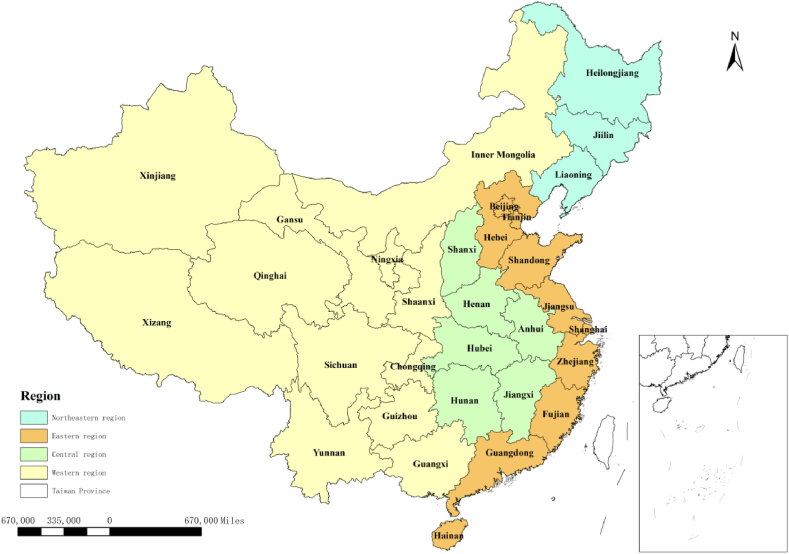
Four regions composed of different provinces in China (Sources: Authors).

Although rapid economic growth has provided a huge opportunity for China, regional inequalities in innovation have also led to many challenges. For instance, based on time-averaged data for 2010 to 2021, the patent authorisation rate in Shanghai, exceeded that of Qinghai province by a ratio of 21:1, indicating that regional disparities are rife in China. Thus, in recent years, efforts have been made to try to make regional economic growth fairer and more efficient [69]. In this study, we examine the spillover of skilled migration at regional and national levels. According to the *Several Opinions on Promoting the Rise of the Central Region of the Central Committee of the Communist Party of China and the State Council, Implementation Opinions of the State Council on Several Policy Measures for Western Development, and Report of the* 16th *National Congress of the Communist Party of China*, China's economic regions are geographically divided into four major regions: eastern, central, western, and northeastern (Fig. 1). This classification can scientifically reflect the social and economic development status of different regions in China, and provide a basis for the Party Central Committee and the State Council to formulate regional development policies. Exploring the spatial spillover effects of skilled migration can provide a useful point of reference for the promotion of innovation capability and regional economic growth. More importantly, this study can help us to understand how to reduce regional inequalities, while promoting innovation efficiency.

### 3.2 Data

Different from previous studies which view GDP as the proxy of economic output, we use innovation capabilities as the measure of growth and our dependent variable. As a measure of innovation capabilities, we selected data on patents granted from 2006 to 2020 in China, which provided us with a measurement of investment in innovation that can cover all the regions over the statistical period [70,71]. Patents can also be regarded as a measure of innovation output [72], and thus can reflect innovation capabilities. In addition, patents measure is effective at an aggregated level, like nation and provinces, because patenting is closely linked to inventive activities overall. Therefore, we measured innovation mobility based on patent data. The data are from statistical yearbooks of various provinces in China, and data at national level are obtained by aggregating data from each province. With the development of market economy, there are increasing enterprises operating across regions. This leads to the possibility of duplicate statistics in the locations of subsidiaries and parent companies. In addition, the differences in economic development among different regions make it difficult to establish unified statistical standards. Only statistical principles and range ranges can be established. Therefore, the basic information used nationally and locally is not entirely consistent. In order to investigate the spatial spillover effects of skilled migration on innovation at different spatial scales, we choose local statistical yearbooks, rather than Chinese statistical yearbooks.

Following the studies of Liu and Shen [9], and Gu and Shen [8], skilled migration refers to individuals with tertiary education or above as their highest education degree, and they reside in a province of usual residence different from 5 years ago. The samples in the study only include individuals who were aged from 24 to 64. The data source of this variable is the part of population educational degree in tabulation population census of the People's Republic of China. Based on different perspectives, the data of the National Statistical Yearbook is the result of evaluating and modifying initial data from various regions. It is more suitable for national level analysis. However, it does not reflect the demographic information required in this article. Therefore, this data source of tabulation population census is reliable.

### 3.3 Method

The study considers the analysis model where skilled migration shows up as the improvement of regional innovation capabilities [73]. The change of skilled migration in a region is a basic innovation measurement, which could help to open up a new industry. The identification of skilled migration with regional innovation capabilities can be viewed as a predictor of long-term growth. In addition, the progress in innovation capabilities shows up as the quality and quantity improvement for labour [74,75]. The improvement indicates the continuous process of innovation-led industry upgrading. Thus, a common point in the literature is to begin from a stylised regional production function to model the transition approaches of production factors to economic growth [76]. Meanwhile, the function is usually developed by a spatial extension [77]. The study uses Cobb-Douglas (C–D) production function as baseline to estimate the effect of skilled-migration in innovation output. In theory, this model is widely used to predict the production of industrial systems or large enterprises in countries and regions, and to analyse and develop production pathways [78]. This model shows that the main factors determining the level of industrial system development are the input of labour, fixed assets, and technology or knowledge, and it can detect three types of growth [79]. The first type is the incremental reward type, indicating that expanding production scale based on existing technology and knowledge is beneficial for increasing output. The second is the diminishing return type, indicating that using existing technology and knowledge to expand production scale is not worth the loss. Finally, the constant reward type indicates that production efficiency will not increase with the expansion of production scale. Only by improving technological and knowledge level can economic benefits be improved. Therefore, the model emphasizes the role of technological innovation and knowledge in the final output. Empirically, this model has also been widely applied to analyse the impact of technological innovation [80], skilled labour [81], knowledge-based transformation of industrial structure [82], and innovation agglomeration [83] on regional and national growth. At equilibrium, the empirical model within the C–D production function (Equation (1)) is established as:

(1) $$Y=A\left(t\right){\;L}^{\alpha }\;K^{\beta }\;\mu$$

Where, Y is output/regional development. L is labour input, and connecting to our study, the data is except skilled labour. K is capital stock; A(t) is comprehensive technology level; α is the elasticity coefficient of labour output, and β the elasticity coefficient of capital output. μ indicates the effect of random interference, and μ ≤ 1.

Following previous studies [84,85], the study employs an aggregate C-D production function at different spatial scales to link regional skilled migration with regional development (innovation capabilities). Thus, the primary regression model can take the following form by log-linearised reduced version:

(2) $$\ln\;IC=\beta_{0}+\beta_{1}\;\ln\;L+\beta_{2}\;\ln\;PC+\beta_{3}\;\ln\;GC+\beta_{4}\;\ln\;SM+\varepsilon$$

Where, IC denotes regional development measured by regional innovation capabilities; PC and GC are private sector capital stock^1^ and public capital stock,^2^ respectively; SM means skilled migration; ε is error term. Table 1 shows the summary statistics of all the variables. SM presents the characteristic of the zero values, which led us to exclude this part of the data. The exclusion of zero values from equation (2) does not affect the accuracy of the regression results since they represent a small percentage of the sample size. In addition, our study considers the zero values as no mobility between regions, and thus they are out of our aim and data that should be analysed in empirical strategy. Furthermore, the implementation of logarithmic treatment also further ensures the smoothness of the results. Therefore, we exclude the zero values. In addition, connection to our study, the statistics of L and SM may be related, which can result in multicollinearity issues in regression analysis. Multicollinearity means that the model estimation is distorted or difficult to estimate accurately due to the accurate correlation or high correlation between the explanatory variables in the linear regression model. Generally, deleting unimportant independent variables is an effective way to solve the problem. However, when independent variables from the model are deleted, it should be noted that it is the actual economic analysis that helps to determine which variables should be kicked out rather than artificially forced elimination. If the deletion is improper, the model setting error will occur, resulting in serious bias in parameter estimation. Therefore, in order to avoid this issue, this research focuses on the repeated calculation and repeated measurement of variables in the initial process of variable selection and measurement. Furthermore, in order to ensure that the multicollinearity does not affect the estimation results, this thesis conducts a variance inflation factor (VIF) test. According to Table 1, all the test results show that the values of VIF are less than 10. Thus, multicollinearity is not a challenge in our study.

**Table 1:** Basic statistics for variables.

Variables	Min	Max	Mean	SD	VIFs
L	1944	96922	2400.45	879.24	1.29
PC	70.4	78026.6	1179.11	786.56	3.07
GC	17.45	14105.05	2063.06	809.29	2.43
SM	0	193876	5432.37	7432.63	6.31
IC	81	872209	9313	10882.36	1.66

Then, the study considers spatial autocorrelation issues by implementing Moran's I test. Because we plan to explore the spatial spillover effects of skilled migration, the influence of a change in neighbouring and their independent and dependent variables should be considered [86]. Whether spatial autocorrelation is significant can indicate the rationality of ordinary least squares regression. Significant Moran's *I* statistic suggests the data are influenced by their neighbours or spatial autocorrelation, which shows the necessary of spatial regression. Regarding this kind of model, in addition to the spatial lag of endogenous variables, this kind of econometric model contains the spatial lags of all the exogenous variables. Thus, referenced from LeSage and Pace [87], we construct a Spatial Durbin Model (SDM) for the analysis. The SDM was a static model because it did not consider the temporal autocorrelation between different time periods, but it can help to emphasize spatial effects of skilled migration to regional innovation. Furthermore, different from the combination between general innovative growth model and stochastic production frontier model that is more suitable for analysis at the micro enterprise level [88], SDM is more inclined towards macro analysis at the integration level. In addition, although the SDM neglects to examine temporal autocorrelation, it will also provide empirical evidence for the combination of labour and agglomeration economies by considering spatial lag variables. Therefore, SDM can directly help us answer the spatial effects of skilled migration in regional growth.

(3) $$IC=\rho WIC+\sum_{k}\beta_{k}X_{k}+\sum_{k}\lambda_{k}WX_{k}+\alpha \nu_{n}+ℇ$$

Where, ρ and W are spatial autocorrelation coefficient and spatial weight matrix, respectively; X denotes a set of k control variables; $\nu_{n}$ indicates an n×1 vector of ones; β, λ and α show the vectors of regression coefficient estimation; ℇ means the error term, and k is the number of independent variables. Thus, based on LeSage and Pace [87], the spatial lag of the dependent variable and the spatial lagged explanatory variables are WIC and $\sum_{k}\lambda_{k}WX_{k}$ respectively.

Furthermore, we construct form SDM framework. The change of a dependent variable in a single region could influence other regions by network effects, and the change of independent variables in a single observation would potentially influence all other observations [85]. Thus, based on equations (2), (3), the key estimation model (Equation (4)) for spatial spillover effects of China's skilled migration can be established as follows:

(4) $$\ln\;{IC}_{it}=\rho \sum_{j=1}^{n}w_{ij}\;\ln\;{IC}_{jt}+\beta_{0}+\beta_{1}\;\ln\;L_{it}+\beta_{2}\;\ln\;{PC}_{it}+\beta_{3}\;\ln\;{GC}_{it}+\beta_{4}\;\ln\;{SM}_{it}+\theta_{1}\sum_{j=1}^{n}w_{ij}\;\ln\;L_{jt}+\theta_{2}\sum_{j=1}^{n}w_{ij}\;\ln\;{PC}_{jt}+\theta_{3}\sum_{j=1}^{n}w_{ij}\;\ln\;{GC}_{jt}+\theta_{4}\sum_{j=1}^{n}w_{ij}\;\ln\;{SM}_{jt}+\varepsilon_{it}$$

Where, i is province and t means time (year); j is nearby provinces, and j≠i. Within an SDM framework, the regional variation in patent counts levels is modelled to depend on the patent counts levels from the neighbouring provinces captured by the spatial lag vector WIC, as well as the factors input of neighbouring provinces represented by $\sum_{k}\lambda_{k}WX_{k}$. Therefore, our main estimation parameter are the coefficients of L, PC, GC, SM, and their spatial weight coefficients, as well as ρ. A positive value indicates a positive effect, while a negative value indicates a negative impact. Whether this effect is significant depends on the *p*-value. In addition, a binary contiguity matrix is chosen to construct spatial weighted matrix [85]. The weight matrix (Equations (5), (6))) in the study is in row-standardization. It can effectively eliminate biased estimates generated in linear regression, thereby improving the performance of spatial weight matrix regression [89]. In addition, it can also ensure consistency in the impact of sample size on the results and help to test the effect of adjacent areas [90]. The method assumes only contiguous provinces can affect each other:

(5) $$w_{ij}=\left\{ \begin{array}{c} \;1\;i\neq j\\ 0\;i=j \end{array}\right.$$

(6) $$\sum_{j=1}^{n}w_{ij}=1$$

It is noteworthy that Hainan Province, as an island, does not appear to be in close proximity to any of the provinces. However, as early as the early 20th century, the Chinese government took this into account by connecting it to Guangdong Province through railway transport. The socio-economic development of Hainan Province was also incorporated into Guangdong Province. Based on this, when constructing the weight matrix $w_{ij}$, Hainan Province was set to be connected to Guangdong Province only.

Next, we examine the direct, indirect and total impacts. Provincial borders are chosen to indicate spatial geographic unit. Evaluation at the provincial level enables the results from the SDM to be directly explained in the process of interpreting the territory. Meanwhile, it is reasonable to conduct the change from regional to provincial level, because the complexity in China's provinces is the same as their regions regarding their social relationships, quality and kinds of resources [91]. Governments at a provincial level have the powers to decide on innovation input, which is necessary for policy implications. Based on LeSage and Pace [87], this study calculates the direct, indirect and total impacts within the context of SDM to better understand spatial spillovers. The measurements focus on the accumulative effects of skilled intern migration, leading to the change in the long-run steady-state equilibrium [85]. The aim of the measures is to explore whether the increasingly positive effects of skilled migration in a region are accompanied by negative spillover from other regions.

Furthermore, because of linearity and the assumed independence of observations, linear regression parameters can be viewed simply as the partial derivation of a dependent variable to the explanatory variable [76]. SDM expands the information set by including all information from neighbouring regions or observations. Thus, we consider the SDM (Equations (7), (8), (9), (10))) as:

(7) $$\left(I_{n}-\rho W\right)IC=\alpha +X\beta +WX\theta +\iota_{n}+\varepsilon$$

(8) $$IC=\sum_{r=1}^{k}S_{r}\left(W\right)x_{r}+V\left(W\right)\iota_{n}\alpha +V\left(W\right)\varepsilon$$

(9) $$S_{r}\left(W\right)=V\left(W\right)\left(I_{n}\beta_{r}+W\theta_{r}\right)$$

(10) $$V\left(W\right)={\left(I_{n}-\rho W\right)}^{-1}$$

From a region to n regions, Equation (8) can be re-written as the following matrix form (Equation (11)):

(11) $$\left(\begin{array}{c} \begin{array}{c} {IC}_{1}\\ {IC}_{2} \end{array}\\ \vdots \\ {IC}_{n} \end{array}\right)=\sum_{r=1}^{k}\left(\begin{array}{ccc} \begin{array}{c} S_{r}{\left(W\right)}_{11}\\ S_{r}{\left(W\right)}_{21} \end{array} & \begin{array}{c} S_{r}{\left(W\right)}_{12}\\ S_{r}{\left(W\right)}_{22} \end{array} & \begin{array}{c} \begin{array}{cc} \cdots & S_{r}{\left(W\right)}_{1n} \end{array}\\ \begin{array}{cc} \cdots & S_{r}{\left(W\right)}_{2n} \end{array} \end{array}\\ \vdots & \vdots & \ddots \\ S_{r}{\left(W\right)}_{n1} & S_{r}{\left(W\right)}_{n2} & \begin{array}{cc} \cdots & S_{r}{\left(W\right)}_{nn} \end{array} \end{array}\right)\left(\begin{array}{c} \begin{array}{c} x_{1r}\\ x_{2r} \end{array}\\ \vdots \\ x_{nr} \end{array}\right)+V\left(W\right)\varepsilon$$

Where, the derivative of ${IC}_{i}$ with respect to $x_{jr}$ obeys potentially non-zero, and the value is determined by the i and j th element of the matrix $S_{r}\left(W\right)$ (Equation (12)).

(12) $$\frac{\partial {IC}_{i}}{\partial x_{jr}}=S_{r}{\left(W\right)}_{ij}$$

An implication is that the change in an independent variable for a single region could potentially impact the dependent variable in other regions. That is also the condition that the derivative of ${IC}_{i}$ regarding $x_{ir}$ does not equal $\beta_{r}$ as in least-squares (Equation (13)):

(13) $$\frac{\partial {IC}_{i}}{\partial x_{ir}}=S_{r}{\left(W\right)}_{ii}$$

The own derivative in the i th region examines the impacts on the dependent variable observation i from the change in $x_{ir}$. Therefore, we will also calculate some new parameters as follows. The average of diagonal elements of matrix $S_{r}\left(W\right)$ can help to obtain average direct impacts, and the sum of the rows average of matrix $S_{r}\left(W\right)$ over all regions can help to obtain the average total impacts. The average indirect impacts (spillover effects) (Equations (14), (15), (16))) are the difference between total effects and indirect effects. In equations:

(14) $$\bar{M}\left(r\right)total=n^{-1}\iota_{n}^{\prime }S_{r}{\left(W\right)}_{\iota_{n}}$$

(15) $$\bar{M}\left(r\right)direct=n^{-1}tr\left(S_{r}\left(W\right)\right)$$

(16) $$\delta =\bar{M}\left(r\right)indirect=\bar{M}\left(r\right)total-\bar{M}\left(r\right)direct$$

Where, δ is spatial spillover. Subtracting the average indirect effect from the total effect yields the spatial spillover effect because this portion of the influence is the result of indirect transmission through adjacent areas rather than directly affecting the target area. Therefore, we examine the spatial spillover effects (μ or indirect impacts) of skilled migration at national and regional levels. This analysis helps us understand how and to what extent different regions influence each other spatially.

Considering the endogeneity issues, the study selects Maximum Likelihood (ML) estimation. Alternatively, Generalised Method of Moment s (GMMs) is also used to deal with endogeneity. However, it includes spatially lagged independent variables, which cannot help us to explore the impacts of spatial spillover [92]. Thus, we implement ML procedures.

## 4 Results and discussion

### 4.1 Spatiotemporal patterns of skilled migration

**Fig. 2:**
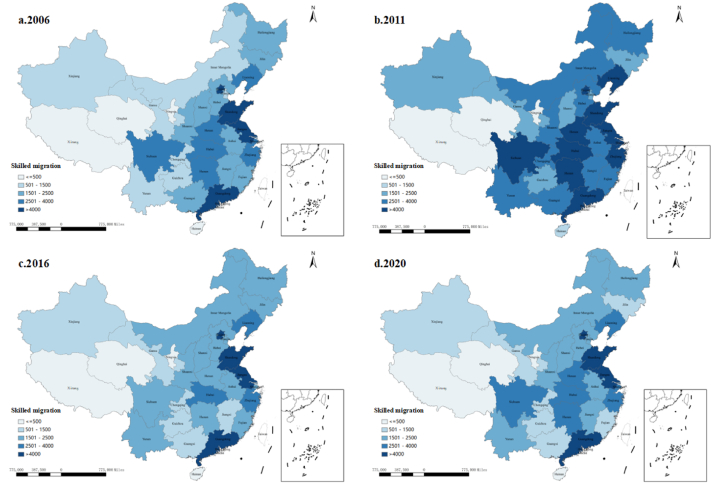
The spatial patterns of skilled migration from 2006 to 2020 (Sources: Authors).

Because the official economic planning in China usually takes five years as a time period, this study divides the statistical period into three sub-periods: 2006–2010, 2011–2015, and 2016–2020, to explore and compare the spillover effects changes over time. Based on the population data on higher education in provincial statistical yearbooks, Fig. 2 demonstrate the spatiotemporal distribution of skilled migration from 2006 to 2020. According to Fig. 2(a–d), although skilled migration has gained a significant increase since 2006, its spatial pattern is highly imbalanced and its spatial agglomeration could be found during this time period. Overall, the group of rich skilled migration tends to cluster a long the coast of China, which demonstrates the positive association between agglomeration and regional growth [93]. Based on endogenous growth theory, spatial spillover effects would result in the concentration of economic activities in neighbours because of effective access to innovation and transport [94]. Thus, increasing agglomeration of skilled migration in eastern coast China can contribute to the agglomeration and growth in central region. The groups of poor skilled migration are clustered in northeastern and western China. Moreover, the northeastern region witnesses an overall decrease over time. The spatial agglomeration of these weak regions further increases regional inequalities because of little skilled labour.

From the temporal perspective in Fig. 2(a–d), the clusters of skilled migration are increasing over time. The results indicate that economic agglomeration in skilled migration is shaping China's economic geography. However, the positive spatial effects of agglomeration economy have not been fully developed, and spatial inequalities still exist because some backward regions are still located between developed regions. For example, although its neighbouring provinces have increased, Hunan province always belongs to the poorest skilled migration level. This may result from the absence of growth poles, transportation costs, and ecology-fragile regions [69]. In addition, the localised clustering of provinces with little skilled migration tends to decrease by some poor provinces getting more policy support or investment attraction. With the regional economy developing fast, the regional inequalities across provinces tend to be decreasing in the long term, and the role of skilled migration agglomeration in growth will be further expanded.

### 4.2 Results on spatial spillover effects at national and regional levels

Table 2 shows the estimation results of SDM at the national level. Overall, the labour, two kinds of capital and skilled migration are significantly positive for innovation capabilities. In spatial models, compared with R^2^, a smaller absolute value of Log-likelihood reflects the goodness-of-fit of the SDM model. Table 2 show sound Log-likelihood in all models. As the spatial autocorrelation coefficient, ρ is significant and positive, meaning that China is characterised by a positive and significant spatial correlation. Regarding the spatial lagged explanatory variables, the output of national innovation capabilities is a positive function with respect to labour, private capital and skilled migration, whereas the public capital is negative. By infrastructure and tertiary education, public capital can lead to spillover effects, which will have indirect impacts on innovation capabilities [95]. Lack of infrastructure and low quality of tertiary education will reduce the effects of public capital, and public capital cannot effectively stimulate innovation ability [96]. Thus, in view of the current situation of infrastructure construction and educational progress in China, the positive effects of public capital may not have been highlighted. In addition, the vicious competition between horizontal and vertical governments makes the spatial spillover of public capital have a negative impact on innovation output.

**Table 2:** Estimation results of SDM at the national level.

Variables	2006-2020	2006-2010	2011-2015	2016-2020
Constant	1.283	0.154***	1.125**	0.451***
L	0.007***	0.071**	0.007*	0.023***
PC	0.137***	0.001**	-0.022	0.029***
GC	0.028**	0.055***	0.027***	0.061***
SM	0.017***	0.141***	0.267***	0.021***
ρ	0.004**	0.018***	0.047***	0.005***
W*L	0.030**	0.011**	0.005***	0.006
W*PC	0.012***	0.085***	0.017***	0.048***
W*GC	-0.044	-0.215	-0.026*	-0.007
W*SM	0.107***	0.262***	0.010**	0.178***
R^2^	0.667	0.538	0.712	0.629
Log Likelihood	189.37	166.52	174.16	151.33

Note:

*Statistical significance at the 10% level

**Statistical significance at the 5% level

***Statistical significance at the 1% level. W*variable means spatial lagged independent variables.

In order to offer the accurate spillover effects, especially skilled migration, the study indicates the direct, indirect and total effects of explanative variables in Table 3. The empirical results demonstrate that the total effects of labour (L) are positive and significant for the whole statistical period, and the output elasticity is 0.054 at the 5 % level. The existing studies have argued that labour could favour innovation in the following ways: reduction in the reallocation of labour from declining and old sectors to new and dynamic ones [97], appropriate rents from innovation by higher wage claims [98], increasing rates of job turnover, and people with new networks and new ideas [99]. Thus, the increase in the number of labour provides a great possibility for China's innovation and growth [100]. In terms of capital, both private capital and public capital are significant and positive signs at 1 % and 5 % levels respectively, over the whole statistical period, and private capital has better effects (0.329). These results indicate that firms or private enterprises are the main driving force of innovation in China. Moreover, after China's economic reform, marketisation and globalisation have promoted innovation output and growth by the role of private capital.

**Table 3:** The direct, indirect and total effects of independent variables.

Variables		2006-2020	2006-2010	2011-2015	2016-2020
L	Direct effects	0.016**	0.142*	0.043**	0.074**
	Indirect effects	0.038*	0.094**	0.067*	0.025*
	Total effects	0.054**	0.236***	0.110**	0.099***
PC	Direct effects	0.221**	0.053*	0.103***	0.066*
	Indirect effects	0.108**	0.091**	0.049*	0.095**
	Total effects	0.329***	0.144**	0.152***	0.161**
GC	Direct effects	0.213**	0.127***	0.171*	0.555**
	Indirect effects	-0.074	-0.067	-0.046	-0.194
	Total effects	0.139**	0.060***	0.125*	0.361**
SM	Direct effects	0.462**	0.049*	0.083***	1.091***
	Indirect effects	0.291***	0.137**	0.029*	0.629**
	Total effects	0.753***	0.188**	0.113***	1.720***

Note:

*Statistical significance at the 10% level

***Statistical significance at the 5% level

****Statistical significance at the 1% level.

Considering the impact of skilled migration (SM) in Table 3, the study finds that SM has positive total effects on national innovation output (0.753). Moreover, the influences have been increasing over time because the direct effects of SM rise from 0.049 during 2006–2010 to 1.091 during 2016–2020. Generally, skilled migrants are concentrated in technology-intensive sectors, which are more productive and larger, and generate a higher value-added and a higher profit and capital [101]. Moreover, the migration of skilled individuals is considered as a crucial mechanism of innovation transfer and diffusion because they can enrich the local knowledge by the higher level of productivity and creativity of local interactions, as well as the increasing availability of valuable human capital [1]. In addition, the increasing effects of SM over time can be a result of transportation investment increases [85], meaning that labour mobility is more flexible, and has continuously increased since China's market economic reform. In terms of indirect effects of SM, the spatial spillover is 0.219 with 1 % significance, indicating that skilled migration can polish up innovation output both directly and indirectly through regional spillover effects. Furthermore, the spatial spillovers (indirect effects) of SM are significant in all periods and show an increasing trend because the coefficients are 0.137 from 2006 to 2010 and 0.629 from 2016 to 2020. Overall, due to increasing skilled migration, the results in Table 3 depict that the spatial spillover effects are positive and significant for innovation output and the effects are increasing over time. Increasing skilled migration contributes to knowledge diffusion between regions and leads to indirect social and regional externalities because of technological progress and better transport network accessibility. The impacts of skilled migration on innovation are mediated by localised networks and institutions because their interaction may generate, import and diffuse new technologies to a region [102].

Table 4 shows the estimation results of SDM at the regional level. Overall, the elasticities of spatial spillover (shown by δ) are different in different regions over the whole period. According to Table 4, from 2006 to 2020, the values of μ are 0.153 in the eastern region, 0.09 in the northeastern region, −0.051 in the central region and −0.038 in the western region. These elasticities are statistically significant. Regarding the eastern region, the neighbouring SM has a positive effect on innovation output and the elastic coefficient (0.153) is the highest in all regions. Thus, the result means that when the skilled migration in neighbouring regions rises by 1 %, the patent counts in the eastern region will increase by 0.153 %. In every sub-period, the skilled migration in the neighbouring region has positive external effects, and their output elasticities of neighbouring migration are significantly positive with the values of 0.064, 0.165 and 0.141 in Table 4. Due to a higher level of urbanisation, China's eastern region has highly skilled migrants inflow, which would increase the openness awareness and the dynamics of the regional innovation system, and the number of urban highly skilled migration can directly lead to an increase in urban innovation output [103]. Similarly, the value of 0.090 in the northeastern region indicates positive and significant spillover effects. However, the value is lower than China's eastern region (0.153), and thus, the spillover in the northeastern region is weaker than the eastern region. For sub-periods in the northeastern region, the spillover effects show an increasing trend over time (0.011, 0.067 and 0.071 in three periods, respectively), though it was insignificant in 2006–2010. Moreover, the last two sub-periods also present positive externalities. Because of interregional wage differentials, career prospects and the quality of life, the northeastern region is less attractive to skilled immigrants than the eastern region [9]. Due to the national position of industrial base in northeast China, it still maintains a positive spillover effect and externality. Once this position is lost or no policy preference, whether skilled immigrants can still hold a positive effect depends.

**Table 4:** Estimation results of SDM at the regional level (Eastern, Northeastern, Central and Western).

Regions	Variables	2006-2020	2006-2010	2011-2015	2016-2020
Eastern	L	0.010**	0.020***	0.102***	0.052***
	PC	0.018**	0.095***	0.096***	0.049***
	GC	0.021**	0.021***	-0.042**	0.003
	SM	0.031***	0.047***	0.063***	0.121***
	ρ	0.012**	0.033**	0.002**	0.025***
	W*L	0.022**	0.239***	0.019***	0.008***
	W*PC	0.004	0.067***	0.007***	0.083***
	W*GC	-0.007	-0.041**	0.003	-0.101***
	W*SM	0.063***	0.101***	0.242***	0.129***
	δ	0.153***	0.064**	0.165***	0.141***
	R^2^	0.513	0.602	0.554	0.671
	Log Likelihood	137.46	123.59	119.87	141.22
Northeastern	L	0.038***	0.018***	0.017***	0.024***
	PC	0.012***	0.071**	0.088***	0.028***
	GC	0.024	0.006***	0.025***	0.004***
	SM	0.007***	0.086***	0.139***	0.129***
	ρ	0.004**	0.046***	0.016***	0.021***
	W*L	0.018***	0.026***	0.012**	0.006
	W*PC	0.024	0.054***	0.008	0.013**
	W*GC	-0.027	-0.022	-0.021	-0.118***
	W*SM	0.019***	0.142***	0.047***	0.179***
	δ	0.090***	0.011	0.067**	0.071**
	R^2^	0.694	0.701	0.653	0.621
	Log Likelihood	154.32	171.09	131.37	165.92
Central	L	0.011**	0.023***	0.095***	0.082***
	PC	0.019**	0.020***	0.037***	0.051***
	GC	0.005***	0.003**	0.002**	0.026***
	SM	0.023**	0.096***	0.038***	0.130***
	ρ	0.013**	0.067***	0.008***	0.049***
	W*L	0.031***	0.047***	0.241***	0.020***
	W*PC	0.004**	0.239***	0.022***	0.050***
	W*GC	-0.006	-0.001	-0.003***	-0.011
	W*SM	0.063***	0.101***	0.103***	0.121***
	δ	-0.051**	-0.077***	-0.105***	-0.125***
	R^2^	0.692	0.563	0.415	0.508
	Log Likelihood	142.67	153.61	166.52	171.94
Western	L	0.022**	0.052***	0.044**	0.033*
	PC	0.025***	0.054***	0.017**	0.018**
	GC	0.013	0.024**	0.014*	0.011*
	SM	0.051***	0.049***	0.068***	0.138***
	ρ	0.015**	0.079***	0.027***	0.054**
	W*L	0.049**	0.083***	0.020*	0.106**
	W*PC	0.009*	0.121***	0.015*	0.072*
	W*GC	-0.053	-0.006**	-0.046	-0.096
	W*SM	0.083***	0.130***	0.136****	0.173**
	δ	-0.038***	-0.011	-0.077*	-0.065***
	R^2^	0.588	0.509	0.496	0.517
	Log Likelihood	157.92	168.35	173.29	169.51

Note:

*Statistical significance at the 10% level

***Statistical significance at the 5% level

****Statistical significance at the 1% level. W*variable means spatial lagged independent variables.

However, in the central region, the negative coefficient (−0.051) indicates that the inflow of skilled migration may be detrimental to patent output. Through comparing the results for the sub-periods (−0.077, −0.105 and −0.125), we can find that the growth of skilled migration in neighbouring regions is negative for regional innovation capabilities all the time, and the negative effects are increasing over time. Globalisation makes China's ‘factory of the world’ move from the coastal region to the central region, and thus, manufacturing has become the main force of economic growth in the central region [104]. The manufacturing industry has relatively low requirements for technology and knowledge. The value of skilled migration itself is difficult to be applied in this region. Meanwhile, in recent years, inefficient industrial transformation and industrial upgrading in the central region can help us understand the increasingly negative effects of skilled migration. Similarly, the μ value (−0.038) in the western region is also negative. However, this value is larger than that in the central region, meaning that the policy effect of China's western development begins to appear gradually. For sub-periods in the western region, the results of μ show that the spillover effects have been strengthened yearly, as shown in the increasing statistical significance.

### 4.3 Discussion on spillovers change over time

Overall, the empirical results at national and regional levels suggest the significant spatial spillover effects of skilled migration. Moreover, based on different sub-periods, we can also observe the change in spillover between regions over time. In order to deeply understand the regional difference in spillover, we examine how the spatial spillover of skilled migration works in China's regions. Unlike the existing studies that analyse migration spillover from the perspectives of individual perception and demographic characteristics [105,106], this study regards institutional and economic types of spillover effects as the sources of skilled migration spillovers. As the task of economic growth has been more and more detailed to local areas in China, various regions or cities have issued kinds of preferential policies to attract talents. Thus, institutional incentives could result in positive spillover due to urban preferential conditions to skilled migration [107]. On the other hand, the spillovers of skilled migration result from economic factors mobility, such as knowledge, technology, information and socioeconomic network etc. The mobility makes skilled migration gather in economically developed regions, which further exacerbates regional inequalities [108]. Thus, connecting to China's situation, the study understands the changes in spillover effects by institutional preference and economic factor mobility.

Regarding institutional preference, the integration of migration into a host place or society influences a range of policy fields within the jurisdiction of local authorities [109]. Thus, institutions that are used to guide migration are different considerably between territorial units nested within a county or region [110]. Studies on China have long underlined the role of state in regional development, and with the emergence of institutional economics and the institutional turn in economic geography, the way of regional growth relying on institutions has been further strengthened [111]. The state plays a critical role in China's regional economy and development by its effects on growth mechanisms, global cities, entrepreneurial cities and urban governance [112]. In China, the market is incomplete and governed, and the capabilities of the state are reflected by policy instruments and institutional linkages with private enterprises [113]. Similarly, China's economy transition has many forms and following characteristic: recombinant property, political fragmentation and partial and uneven reform of socialist institutions [114]. China's economic transition has fundamentally restructured the socialist state orthodox. Because China's institutions are constantly evolving and transitional, transitional institutions are internally incoherent and unstable, and may encounter the following situations: rent-seeking behaviours, spatial segmentation, conflicts among different organizations etc. [112]. For example, China's state policies have led to the rise of the coastal-interior divide, foreign investment and high-tech industries. Taking Pearl River Delta, the Yangtze Delta, Beijing-Tianjin and Shanghai as the cases, much literature clarifies the significant roles of local states and places in regional development [115,116]. These regions with knowledge agglomeration are expected to produce spatial spillover.

In addition, some economic literature which is closer to the neoclassical economic schools has attempted to establish the linkage between economic output and region-specific institutional structures [117]. These studies focus on how effective institutions deal with the provision of public goods, market failures and labour within a region [118]. Certainly, these works obey the basic assumptions of neoclassical economics, such as completely rational individual behavior [119]. On the contrary, some studies approve that the regional labour market environment not only shape institutions, but also is shaped by institutions. Therefore, institutions are sensitive to places, that is, although institutions share some common features across regions, they also have a place distinctiveness and are fed by local institutional environment [120]. Institutions finally improve economic efficiency by reducing transaction costs [121], promoting entrepreneurship and expanding labour markets [122]. Consequently, specific local institutional arrangements can enable regions to embark on a sustainable and high-end economic development path [118]. Moreover, these institutional arrangements work better at both the local and regional levels, because institutions at the national level may be too remote to effectively address specific regional issues. Meanwhile, endogenous growth theory also indicates that if a region can attract local labor assets and link externalities and scale economies with spatial and specialized clusters, it would produce competitive advantage [123]. Thus, a region has to reduce transaction costs and increase technological innovation. This is the focus of institutional economy, which emphasizes the proximity and association of the regions to a certain centre and reflects a learning and knowledge source.

Furthermore, integration policies help to regulate immigrants' access to the market, mobility and the state [124]. The literature on citizenship policies suggests that state-led countries make it easier for those from different ethnic/cultural backgrounds to join the country [125]. The policies at sub-national level would be more significant to immigration. For China's skilled migration, policy coordination between regions and policy integration between adjacent regions have positively influenced China's regional innovation, especially intellectual property rights institutions [126]. Moreover, the institutional preference not only provides more economic convenience for skilled migrants, but also enables them to enjoy equal political rights and social status. Consequently, there exist positive spillovers at the national level across the whole period and different sub-periods since skilled migration could arise the institutional preference among provinces. Meanwhile, results at the regional level indicate that skilled migration spillover changes over time. One possible explanation is the positive externalities caused by the competition of talent introduction policies among regions.

The changes in mobility of economic factors related to innovation between regions can understand the difference in spillovers of these factors mediated by geography. Regional innovation system clarifies the dynamic complexity of innovation factors [127,128]. Although systemic conditions shape the process of cumulative learning, they go beyond the pure Marshallian agglomeration economies which are based on individuals, ﬁrms, and localised institutional and structural factors [129]. Thus, the spillover of skilled migration to innovation is caused by localised networks consisting of private actors and institutions, because their interaction may affect the possibility of the generation, import and dissemination of new technologies [1]. Moreover, the efficiency of economic factor mobility will increase over time. To explain how economic factor mobility changes over time across the four regions, Table 5 depicts the total trend of the net mobility value of economic factors (R&D investment and skilled labour). The Eastern region has always maintained a strong development trend in regional innovation, whereas the northeastern region gradually lost its dominant position in innovation output, possibly because of the far geographic distance to the developed eastern region. The central and western regions have the same trend of improvement. Especially, the central region has been rising since 2011, catching up with and surpassing the innovation development in the northeastern region. The progress in the western region is also the result of economic factors mobility caused by China's national strategy and policy preferences.

**Table 5:** The mobility of economic factors among regions in various periods.

Period	Eastern			Northeastern			Central			Western		
	R&D	SM	Trend	R&D	SM	Trend	R&D	SM	Trend	R&D	SM	Trend
2006–2010	+290	+387	+	+97	+86	+	−217	−168	–	−92	−115	–
2011–2015	+363	+299	+	−1033	−279	–	+232	+326	+	−984	−458	–
2016–2020	+407	+512	+	−318	−409	–	+301	+139	+	+197	+72	+

Note: ‘+’ is shift-in and ‘-’is shift-out.

Table 6 constructs a Saldo matrix^3^ on the spillover change in different sub-periods in the four regions of China, and the line of Saldo is the sum of Source 1 and Source 2. Skilled migration spillover caused by economic factors mobility has the same trend as production factors flow. Furthermore, skilled migration spillover resulted from institutional preference is always positive in all regions, because the values of ρ are positive in Table 3. Thus, the balanced matrix can be obtained. In the eastern region, there are a great R&D input and skilled immigration, and positive spillovers exist in all sub-periods. In particular, this positive effect is gradually strengthened over time. On the contrary, the Saldo matrix witnesses the fading in the northeast region. From 2006 to 2010, the two sources have positive spillover, but the negative spillover from skilled migration counteracted the positive spillover resulted from innovation input from 2011 to 2015. Even, in 2016–2020, they have the same negative sign. Overall, the central and western regions experience the same Saldo trend. From 2006 to 2010, the two regions have negative spillover. However, in 2011–2015, the Saldo of spillover becomes positive in the central region. Although there are strong negative spillovers of source 2 in the western region in the sub-period, the positive spillover of innovation input has begun to appear. In the last sub-period, central and western show positive spillover, especially central region.

**Table 6:** Spillover changes of China's regions in different sub-periods.

Spillovers	Eastern			Northeastern			Central			Western		
	2006–2010	2011–2015	2016–2020	2006–2010	2011–2015	2016–2020	2006–2010	2011–2015	2016–2020	2006–2010	2011–2015	2016–2020
Source 1	+	+	+	+	+	–	–	+	++	–	+	+
Source 2	+	++	++	+	–	–	–	+	+	–	–	+
Saldo	++	+++	+++	++	0	–	–	++	+++	–	–	++

## 5 Conclusions

By promoting local knowledge intensity and increasing the productivity and creativity of local interactions, skilled migration has been traditionally viewed as an important determinant of innovation capabilities in recipient areas. However, few studies involved in spatial effects of skilled migration from a complete quantitative perspective. Thus, our study direct explored the spatial spillover effects of skilled migration at China national and sub-national levels. Due to the consideration of regional disparities, this will benefit future studies because economies tend to experience varying degrees of innovation capabilities with all aspects of the innovative environment in different regions or economies. Overall, we not only map the spatiotemporal patterns of skilled migration, but also show its spatial effects and their changes overall time at different spatial scales. Moreover, our method also captures spatial externalities and endogeneity issues in the mechanisms. The study also extends the literature on employment and wages of skilled labour in labour economics into the discussion of economic geography.

Our results suggest that skilled migration is associated with increasing innovation output, and has significant spillover. At the national level, there exists a positive spillover of skilled migration all the time. However, the spillover at the regional level varies across different sub-periods. In detail, the positive spillover in the eastern region is always held all the time and is strong and significant. Although the northeastern region also has positive spillover, its spillover intensity is much smaller than that in the eastern region, and it is not significant in every sub-period. In addition, the spillover of skilled migration in the central region is significantly negative, and over time, this negative effect is growing. This conclusion is slightly different from the work of Qiu et al. [88] where developing areas benefit from external knowledge spillovers than developed areas. One possible explanation is that the developing regions have not achieved talent agglomeration, or these regions are not attractive to skilled migration. Interestingly, although the development of the western region is backward and the spatial spillover of skilled migration is significantly negative, the degree of this negative spillover is decreasing yearly. In addition, through arguing that the spatial spillover effects of skilled migration are from two sources of institutional preference and mobility of economic factors related to innovation, we understand the changes of spillover effects among Chinese regions. The findings show that although the eastern region has always maintained strong momentum, the northeast region has gradually declined over time. The central region does not benefit from the positive spatial spillover effects of skilled migration in the early stage, but in the middle and late stage, it attached importance to the spatial benefits of skilled migration for innovation output, and maintained positive momentum. Similarly, the change in the western region is from negative to positive over time, and this positive change did not happen until the last sub-period. This also shows that the strategy of China's western development has begun to produce the desired result. Meanwhile, the results on the changes of spillover effects also argue that innovation output resulting from skilled migration in the same region can be at the expense of other regions because the negative spillovers from R&D input and SM factor offer specific evidence.

Regarding policy implications, skilled migration as an approach to foster innovation capabilities should be regarded as the core of policy agenda at different levels. Cross-regional skilled migration should be encouraged because of the existence of spatial externalities. Moreover, the central government should promote regional integration and support skilled immigrants in backward areas. In addition, progress at the expense of neighbourhood interests may exacerbate vicious competition in talents attraction among regions. Thus, each region should establish a reasonable and orderly immigration policy according to the actual situation. Due to the gradual decline of Northeast China, it needs rapid industrial upgrading by favourable skilled immigration policies. Due to its geographical proximity, the central region should continue to attract more skilled migration by absorbing the positive spillover effect of the eastern region. In terms of the current situation and the foreseeable future, the improvement in the innovation ability of the western region still depends heavily on policy support.

However, while the basic task in the study is that skilled migration impacts regional innovation or regional development, an important caveat to our study is that there may exist some forms of endogeneity. For example, a highly innovative region (for whatever reason) also will in turn demand more skilled migration, evolve in a more economically egalitarian fashion, and grow faster (and thus be able to afford more skilled migration) [130]. In fact, the endogeneity of regional growth or innovation and skilled migration has been a major focus of the economic geography and institutional literature. On the one hand, it is the inflows of talents with specific sorting incentives that set off the subsequent rise of regional innovation. On the other hand, it is the underlying switching of productive systems (or innovation) that “attracts” skilled migrants. Our study echoes with the former line and still follows a neoclassical fashion. Although skilled labour is essential in growth models, homogenizing skilled migrants with other general skilled labour in a neoclassical model may completely ignore the spatial sorting incentives that may make the case interesting and relevant to economic geography. In addition, maximum likelihood estimation is employed to assess the endogeneity issues to understand the mechanisms of spillovers change over time. However, maximum likelihood is an estimation method, it per se is not possible to solve the endogeneity issue alone. Thus, future studies could consider thinking or critiques on general spatial equilibrium framework to construct theoretical arguments. In empirical, instrument variable is a better approach to deal with this issue. Furthermore, based on our theoretical explanation, future studies can further calculate the physical spatial range of spatial spillover effects of skilled immigrants. By considering the temporal autocorrelation and comparing the calculated spatial range with the regional economic spatial structure in different periods, we can examine whether the current regional economic space based on administrative division is reasonable. The spatial evolution of regional economy can also be observed. In addition, the radiation range can help policy makers or urban planners reconstruct regional development space, so as to delimit new regional economic growth poles, metropolitan areas or city alliances.

## 6 Data statement

The datasets generated or analysed during this study are available from the corresponding author on reasonable request.

## CRediT authorship contribution statement

**Xing Gao:** Writing – original draft, Methodology, Formal analysis, Data curation, Conceptualization. **Jin Zhu:** Writing – review & editing, Formal analysis, Conceptualization. **He Zhu:** Writing – review & editing. **Xingman Zhang:** Data curation.

## Declaration of competing interest

The authors declare that they have no known competing financial interests or personal relationships that could have appeared to influence the work reported in this paper.

## Footnotes

1 It refers to the capital owned by the individual owner of the capital. The data come from fixed assets investment data of state-owned enterprises, collective enterprises and other types of enterprises in provincial statistical yearbooks.

2 It refers to the capital formed by government investment in the public sector. Under the conditions of market economy and public finance, it means the capital of public facilities formed by infrastructure investment, such as the capital formed by fixed assets investment of public sectors such as highways, airports, water supply systems, electricity, gas and telecommunications. It plays a very important role in the development of the national economy. The data are from the Public Finance section in statistical yearbooks.

3 It originates from the Saldotheorie in law. It is necessary to first clear the opposite return requests in the bilateral contract, and the difference (positive) thus obtained is the unjust enrichment to be returned. The return request of both parties is only an independent part to determine the final difference, and there is only one actual unjust enrichment Cause of action, which is the difference automatically obtained through this liquidation, so there is no need to return the obligor to claim offset. Therefore, the content of the unjust enrichment cause of action of the party who has lost the benefit of the payment is the difference between the amount of his treatment of the payment and the value of the other party's payment. The difference is named for this. If the difference is negative (the value of the received benefit is higher than the amount to be paid) or zero, not only does the counterpart not have to bear the obligation to repay the value, but they also do not have to bear the obligation to repay the value.

## References

[1] Gagliardi L. (2015). Does skilled migration foster innovative performance? Evidence from British local areas. Pap. Reg. Sci..

[2] Asheim B.T., Gertler M.S. (2006).

[3] C.C. Fan, China on the Move: Migration, the State, and the Household. Routledge, London.

[4] Wu C., Ren F., Ye X., Liang X., Du Q. (2019). Spatiotemporal analysis of multiscale income mobility in China. Appl. Geogr..

[5] Sharma M. (2017). Change and continuity of income divide in the American Southeast: a metropolitan scale analyses, 2000–2014. Appl. Geogr..

[6] Zhang W., Bao S. (2015). Created unequal: China's regional pay inequality and its relationship with mega-trend urbanization. Appl. Geogr..

[7] Zhu J., Tang W. (2018). Conflict and compromise in planning decision-making: how does a Chinese local government negotiate its construction land quota with higher-level governments?. Environ. Urbanization.

[8] Gu H., Jie Y., Li Z., Shen T. (2021). What drives migrants to settle in Chinese cities: a panel data analysis. Appl. Spatial Analysis.

[9] Liu Y., Shen J. (2014). Spatial patterns and determinants of skilled internal migration in China, 2000–2005. Pap. Reg. Sci..

[10] Cui C., Wang Y., Wang Q. (2022). The interregional migration of human capital: the case of “first-class” university graduates in China. Appl. Spatial Analysis.

[11] Cantos P., Gumbau‐Albert M., Maudos J. (2005). Transport infrastructures, spillover effects and regional growth: evidence of the Spanish case. Transport Rev..

[12] Dustmann C., Glitz A., Frattini T. (2008). The labour market impact of immigration. Oxf. Rev. Econ. Pol..

[13] Manacorda M., Manning A., Wadsworth J. (2012). The impact of immigration on the structure of wages: theory and evidence from Britain. J. Eur. Econ. Assoc..

[14] Ouyang Y., Huang J., Chen X. (2020). Research progress and trends of geographies of migration from the perspective of “cultural turn”. Prog. Geogr..

[15] Fan C.C., Sun M., Zheng S. (2011). Migration and split households: a comparison of sole, couple, and family migrants in Beijing, China. Environ Plan A.

[16] Wu F. (2010). How neoliberal is China's reform? The origins of change during transition. Eurasian Geogr. Econ..

[17] Chan K.W., Wan G. (2017). The size distribution and growth pattern of cities in China, 1982–2010: analysis and policy implications. J. Asia Pac. Econ..

[18] Lin G.C.S., Wang C.C., Zhou Y., Sun Y., Wei Y.D. (2011). Placing technological innovation in globalising China: production linkage, knowledge exchange and innovative performance of the ICT industry in a developing economy. Urban Stud..

[19] Hsu C.-C., Chien F. (2023). The impact of high economic growth and technology advancement on extensive energy production in China: evidence using NARDL model. Environ. Sci. Pollut. Res..

[20] Zhang J., Zhang K., Zhao F. (2020). Research on the regional spatial effects of green development and environmental governance in China based on a spatial autocorrelation model. Struct. Change Econ. Dynam..

[21] Findlay A.M., Stockdale A., Stewart E. (2002). Professional and managerial migration from core to periphery: the case of English migration to Scottish cities. Int. J. Popul. Geogr..

[22] Yang Z., Pan Y. (2020). Human capital, housing prices, and regional economic development: will “vying for talent” through policy succeed?. Cities.

[23] Henning M. (2020). Regional labour flows between manufacturing and business services: reciprocal integration and uneven geography. Eur. Urban Reg. Stud..

[24] Singhal K., Singhal J. (2022). Technology, knowledge, and manufacturing before the industrial revolution. Prod. Oper. Manag..

[25] Gould E.D. (2019). Explaining the unexplained: residual wage inequality, manufacturing decline and low-skilled immigration. Econ. J..

[26] Chongvilaivan A., Hur J. (2019). Structural change and relative demand for skilled workers: new evidence from the US manufacturing. Ind. Corp. Change.

[27] Zeng Z., Wang S. (2009).

[28] Caselli F., Coleman W.J. (2002). The U.S. Technology frontier. Am. Econ. Rev..

[29] Fan L. (2009). Measuring interprovincial flows of human capital in China: 1995–2000. Popul Res Policy Rev.

[30] Fan C.C. (2005). Modeling interprovincial migration in China, 1985-2000. Eurasian Geogr. Econ..

[31] Simon C.J., Nardinelli C. (2002). Human capital and the rise of American cities, 1900–1990. Reg. Sci. Urban Econ..

[32] Yang J., Zhang M. (2021). The value of entrepreneurship and the entrepreneurial ecosystem: evidence from 265 cities in China. Growth Change.

[33] Zhang C. (2020). Skill diversity of cities and entrepreneurship. Reg. Stud..

[34] Jin C., Li B., Jansen S.J.T., Boumeester H.J.F.M., Boelhouwer P.J. (2022). What attracts young talents? Understanding the migration intention of university students to first-tier cities in China. Cities.

[35] Wang L., Xue Y., Chang M., Xie C. (2020). Macroeconomic determinants of high-tech migration in China: the case of Yangtze River Delta urban agglomeration. Cities.

[36] Wei Y.H.D., Liefner I. (2012). Globalization, industrial restructuring, and regional development in China. Appl. Geogr..

[37] Wang G.Y. (2022). Talent migration in knowledge economy: the case of China's silicon valley, Shenzhen. Int. Migration & Integration.

[38] Zhai K., Moskal M. (2022). The impact of place of origin on international and domestic graduates' mobility in China. Int. Migrat. Rev..

[39] Rijkers B., Söderbom M., Loening J.L. (2010). A rural–urban comparison of manufacturing enterprise performance in Ethiopia. World Dev..

[40] Lucas R.E. (1993). Making a miracle. Econometrica.

[41] Rosenthal S.S., Strange W.C. (2004).

[42] McCann P., Ortega-Argilés R. (2015). Smart specialization, regional growth and applications to European union cohesion policy. Reg. Stud..

[43] Artz G.M., Kim Y., Orazem P.F. (2016). Does agglomeration matter everywhere?: new firm location decisions in rural and urban markets*. J. Reg. Sci..

[44] Zucker L., Darby M. (1998).

[45] Zucker L., Darby M., Brewer M. (1998). Intellectual human capital and the birth of US biotechnology enterprises. Am. Econ. Rev..

[46] Cermeño A.L. (2019). Do universities generate spatial spillovers? Evidence from US counties between 1930 and 2010. J. Econ. Geogr..

[47] Brülhart M., Sbergami F. (2009). Agglomeration and growth: cross-country evidence. J. Urban Econ..

[48] Moretti E. (2004). Human capital externalities in cities. Handb. Reg. Urban Econ..

[49] Jacobs J. (1969).

[50] Crescenzi R., Gagliardi L., Percoco M. (2013). Social capital and the innovative performance of Italian provinces. Environ Plan A.

[51] Iammarino S., Piva M., Vivarelli M., Von Tunzelmann N. (2012). Technological capabilities and patterns of innovative cooperation of firms in the UK regions. Reg. Stud..

[52] Glaeser E.L., Rosenthal S.S., Strange W.C. (2010). Urban economics and entrepreneurship. J. Urban Econ..

[53] Poole J.P. (2013). Knowledge transfers from multinational to domestic firms: evidence from worker mobility. Rev. Econ. Stat..

[54] Castillo V., Figal Garone L., Maffioli A., Rojo S., Stucchi R. (2020). Knowledge spillovers through labour mobility: an employer–employee analysis. J. Dev. Stud..

[55] Braunerhjelm P., Ding D., Thulin P. (2020). Labour market mobility, knowledge diffusion and innovation. Eur. Econ. Rev..

[56] Hamrouni D. (2022). International diffusion of knowledge labor productivity and catching up between North and South. Int. Rev. Econ. Finance.

[57] Marshall A. (1980).

[58] Lucas R. (1998). On the mechanics of economic development. J. Monetary Econ..

[59] Acemoglu D. (1998). Why do new technologies complement skills? Directed technical change and wage inequality. Q. J. Econ..

[60] Timmermans B., Boschma R. (2014). The effect of intra- and inter-regional labour mobility on plant performance in Denmark: the significance of related labour inflows. J. Econ. Geogr..

[61] Eppelsheimer J., Möller J. (2019). Human capital spillovers and the churning phenomenon: analysing wage effects from gross in- and outflows of high-skilled workers. Reg. Sci. Urban Econ..

[62] Drivas K., Economidou C., Karamanis D., Sanders M. (2020). Mobility of highly skilled individuals and local innovation activity. Technol. Forecast. Soc. Change.

[63] Kerr W.R., Lincoln W.F. (2010). The supply side of innovation: H‐1B visa reforms and U.S. Ethnic invention. J. Labor Econ..

[64] Hunt J., Gauthier-Loiselle M. (2010). How much does immigration boost innovation?. Am. Econ. J. Macroecon..

[65] Bratti M., Conti C. (2018). The effect of immigration on innovation in Italy. Reg. Stud..

[66] Kerr S.P., Kerr W., Özden Ç., Parsons C. (2016). Global talent flows. J. Econ. Perspect..

[67] Fassio C., Montobbio F., Venturini A. (2019). Skilled migration and innovation in European industries. Res. Pol..

[68] Nathan M. (2015). Same difference? Minority ethnic inventors, diversity and innovation in the UK. J. Econ. Geogr..

[69] He S., Haifeng Liao F., Li G. (2019). A spatiotemporal analysis of county economy and the multi-mechanism process of regional inequality in rural China. Appl. Geogr..

[70] Bretschger L., Lechthaler F., Rausch S., Zhang L. (2017). Knowledge diffusion, endogenous growth, and the costs of global climate policy. Eur. Econ. Rev..

[71] Weinhold D., Nair-Reichert U. (2009). Innovation, inequality and intellectual property rights. World Dev..

[72] Chen Y., Puttitanun T. (2005). Intellectual property rights and innovation in developing countries. J. Dev. Econ..

[73] Franceschi F., Mariani V. (2016). Flexible labor and innovation in the Italian industrial sector. Ind. Corp. Change.

[74] Kaitila J. (2019). From innovation to labour costs: change of emphasis in Finnish competitiveness policy ideas after the Eurocrisis. Compet. Change.

[75] Kaiser U., Kongsted H.C., Rønde T. (2015). Does the mobility of R&D labor increase innovation?. J. Econ. Behav. Organ..

[76] Tientao A., Legros D., Pichery M.C. (2016). Technology spillover and TFP growth: a spatial Durbin model. International Economics.

[77] Ertur C., Koch W. (2007). Growth, technological interdependence and spatial externalities: theory and evidence. J of Applied Econometrics.

[78] Panhans M.T., Biddle Jeff E. (2020).

[79] Mahaboob B., Ajmath K.A., Venkateswarlu B., Narayana C., Praveen J.P. (2019).

[80] Yuan C., Liu S., Wu J. (2009). Research on energy-saving effect of technological progress based on Cobb–Douglas production function. Energy Pol..

[81] Gitto S., Mancuso P. (2015). The contribution of physical and human capital accumulation to Italian regional growth: a nonparametric perspective. J. Prod. Anal..

[82] Da Silva M.A.P.M. (2023). Cobb–Douglas R&D production function, appropriability and opportunity: effects on R&D, technological progress and knowledge sharing. Econ. Innovat. N. Technol..

[83] Cainelli G. (2008). Spatial agglomeration, technological innovations, and firm productivity: evidence from Italian industrial districts. Growth Change.

[84] Li Y., Liu X. (2018). How did urban polycentricity and dispersion affect economic productivity? A case study of 306 Chinese cities. Landsc. Urban Plann..

[85] Yu N., De Jong M., Storm S., Mi J. (2013). Spatial spillover effects of transport infrastructure: evidence from Chinese regions. J. Transport Geogr..

[86] Anselin L. (2001).

[87] Lesage R.K., Pace R. (2009).

[88] Qiu J., Liu W., Ning N. (2020). Evolution of regional innovation with spatial knowledge spillovers: convergence or divergence?. Netw Spat Econ.

[89] Krisztin T., Piribauer P. (2023). A Bayesian approach for the estimation of weight matrices in spatial autoregressive models. Spatial Econ. Anal..

[90] Kim J.-J., Cho M.-J., Lee M.-H. (2022). An analysis of the price determinants of multiplex houses through spatial regression analysis. Sustainability.

[91] Gao X., Cao M., Zhang Y., Liu Y., Tong H., Yao Q. (2021). Towards sustainability: an assessment of an urbanisation bubble in China using a hierarchical - stochastic multicriteria acceptability analysis - choquet integral method. J. Clean. Prod..

[92] Del Bo C.F., Florio M. (2012). Infrastructure and growth in a spatial framework: evidence from the EU regions. Eur. Plann. Stud..

[93] Van Oort F.G., Burger M.J., Knoben J., Raspe O. (2012). Multilevel approaches and the firm‐agglomeration ambiguity in economic growth studies. J. Econ. Surv..

[94] Gardiner B., Martin R., Tyler P. (2011). Does spatial agglomeration increase national growth? some evidence from Europe. J. Econ. Geogr..

[95] Agénor P.-R., Neanidis K.C. (2015). Innovation, public capital, and growth. J. Macroecon..

[96] Agénor P. (2011). Schooling and public capital in a model of endogenous growth. Economica.

[97] Nickell S., Layard R., Ashenfelter O., Card D. (1997). Handbook of Labour Economics.

[98] Malcomson J.M. (1997). Contracts, hold-up, and labour markets. J. Econ. Lit..

[99] Kleinknecht A., Van Schaik F.N., Zhou H. (2014). Is flexible labour good for innovation? Evidence from firm-level data. Camb. J. Econ..

[100] Sahoo P., Dash K.R., Nataraj G. (2010).

[101] Regev H. (1998). Innovation, skilled labour, technology and performance in Israeli industrial firms. Econ. Innovat. N. Technol..

[102] Evangelista R., Iammarino S., Mastrostefano V., Silvani A. (2002). Looking for regional systems of innovation: evidence from the Italian innovation survey. Reg. Stud..

[103] Lyu L., Sun F., Huang R. (2019). Innovation-based urbanization: evidence from 270 cities at the prefecture level or above in China. J. Geogr. Sci..

[104] Li J., Webster D., Jianming C. (2018). Manufacturing-led peri-urbanisation in central China: the case of Wuhan's Dongxihu district. Int. Dev. Plann. Rev..

[105] Pu Y., Han X., Chi G., Wang Y., Ge Y., Kong F. (2019). The impact of spatial spillovers on interprovincial migration in China, 2005–10. Reg. Stud..

[106] Carson D.A., Carson D.B., Lundström L. (2021). Northern cities and urban–rural migration of university‐qualified labour in Australia and Sweden: spillovers, sponges, or disconnected city–hinterland geographies?. Geogr. Res..

[107] Dufhues T., Buchenrieder G., Sun Z. (2021). Exploring policy options in regulating rural–urban migration with a bayesian network: a case study in Kazakhstan. Eur. J. Dev. Res..

[108] Huber F. (2012). On the role and interrelationship of spatial, social and cognitive proximity: personal knowledge relationships of R&D workers in the cambridge information technology cluster. Reg. Stud..

[109] Manatschal A., Stadelmann-Steffen I. (2013). Cantonal variations of integration policy and their impact on immigrant educational inequality. Comp. Eur. Polit..

[110] Zuber C.I. (2020). Explaining the immigrant integration laws of German, Italian and Spanish regions: sub-state nationalism and multilevel party politics. Reg. Stud..

[111] Wei Y.H.D. (2007). Regional development in China: transitional institutions, embedded globalization, and hybrid economies. Eurasian Geogr. Econ..

[112] Wei Y.D., Leung C.K. (2005). Development zones, foreign investment, and global city formation in Shanghai. Growth Change.

[113] Wade R. (1990).

[114] Grabher G., Stark D. (1997).

[115] Yeung Y. (2006). An emerging development focus from the Pearl River Delta west to western Guangdong: a research Report. Eurasian Geogr. Econ..

[116] Depner H., Bathelt H. (2009). Exporting the German model: the establishment of a new automobile industry cluster in Shanghai. Econ. Geogr..

[117] Hodgson G.M. (1993). Institutional economics: surveying the ‘old’ and the ‘new,’. Metroeconomica.

[118] Streeck W., METZNER E., STREECK W. (1991).

[119] Rodríguez-Pose A. (2013). Do institutions matter for regional development?. Reg. Stud..

[120] Gertler M.S., LEE R., WILLIS J. (1997). Geographies of Economies.

[121] Shi W., Huang Q. (2022). The blockchain technology applied in the development of real economy in jiangsu under deep learning. Comput. Intell. Neurosci..

[122] Giddens A. (1990).

[123] Frank V. (2009). Review of regional economics. Reg. Stud..

[124] Entzinger H., Koopmans R., Statham P. (2000). Challenging Immigration and Ethnic Relations Politics. Comparative European Perspectives.

[125] Hussain A., Miller W. (2006).

[126] Gao X. (2021). The impact of density, distance and division on intellectual property rights protection: the case of Huaihai Economic Zone, China. Int. J. Unity Sci..

[127] Zhang L. (2017). The knowledge spillover effects of FDI on the productivity and efficiency of research activities in China. China Econ. Rev..

[128] Asheim B.T., Coenen L. (2005). Knowledge bases and regional innovation systems: comparing Nordic clusters. Res. Pol..

[129] Iammarino S. (2005). An evolutionary integrated view of Regional Systems of Innovation: concepts, measures and historical perspectives. Eur. Plann. Stud..

[130] Sutton J. (1998).

